# Publisher Correction: The impacts of remote learning in secondary education during the pandemic in Brazil

**DOI:** 10.1038/s41562-022-01420-9

**Published:** 2022-07-01

**Authors:** Guilherme Lichand, Carlos Alberto Doria, Onicio Leal-Neto, João Paulo Cossi Fernandes

**Affiliations:** 1 Department of Economics, University of Zurich, Zurich, Switzerland; 2 Department of Economics, University of Brasília, Brasília, Brazil; 3 Inter-American Development Bank, Washington, DC USA

**Keywords:** Economics, Education

Correction to: *Nature Human Behaviour* 10.1038/s41562-022-01350-6, published online 26 May 2022.

In the version of this article initially published, standard error values were missing from the Panel B section of Table 1 (Standardized test scores), columns (1)–(5). The values (reading as “(0.0001)”) have now been inserted in the HTML and PDF versions of the article.

