# Supplementary material for: The impacts of remote learning in secondary education during the pandemic in Brazil
Source: Nat Hum Behav. 2022 May 26;6(8):1079–86. doi: 10.1038/s41562-022-01350-6 (PMC9391221; doi:10.1038/s41562-022-01350-6)
Supplement: Supplementary file 1 — All supplementary results of the paper. [file 41562_2022_1350_MOESM1_ESM.pdf]

nature portfolio

https://doi.org/10.1038/s41562-022-01350-6

Supplementary information

The impacts of remote learning in secondary education during the pandemic in Brazil

In the format provided by the
authors and unedited

Supplementary Materials
The Impacts of Remote Learning in Secondary Education during the Pandemic in Brazil

Guilherme Lichand, Carlos Alberto Doria, Onicio Leal Neto and João Paulo Cossi Fernandes

April 5, 2022

A. Validation of the proxy for student dropouts

This Section compiles evidence to validate our proxy of student dropouts (high dropout risk, equal to 1 if a student had no math or Portuguese scorecard grades assigned to them in the administrative data for that quarter, and 0 otherwise). Section A.1 documents that our proxy in fact correlated with actual dropouts at the classroom level before the pandemic. Section A.2 then shows that students with missing scorecard grades in 2020 had a much higher probability of not participating in any school activities in Q1/2021. Last, in Section A.3, we correct for measurement directly by using analytic formulas that express the bias as a function of the proportion of false positive and false negatives that arise from the proxy, based on administrative data prior to and during the pandemic.

A.1 Validation of the proxy for previous years

We use administrative data from the São Paulo State Education Secretariat, which includes information on both math and Portuguese scorecard grades and enrollment for all middle- and high-school students within State public schools in 2019. Concretely, we define actual dropouts equal to 1 if a student was enrolled in a State school in 2019 but not in 2020, and 0 otherwise. We restrict attention to 6th-11th graders, as we cannot compute actual dropouts for high-school seniors (most of whom disappear from the data because they graduated, not because they dropped out).

Supplementary Figure A.1 plots the prevalence of actual and proxy dropouts at the classroom level, for the universe of 6th-11th graders of São Paulo State. Even though actual dropouts are measured with error – as students might not re-enroll for alternative reasons, from moving to a different State to switching over to a private school –, the figure showcases that the classroom-level actual and proxy dropouts are highly correlated, with a coefficient of approximately 0.7. Since measurement error tends to attenuate this correlation, the coefficient represents a lower-bound to the actual prediction power of this proxy.

Figure S.A.1: Scatter plot of student dropouts (actual) and high dropout risk (proxy)

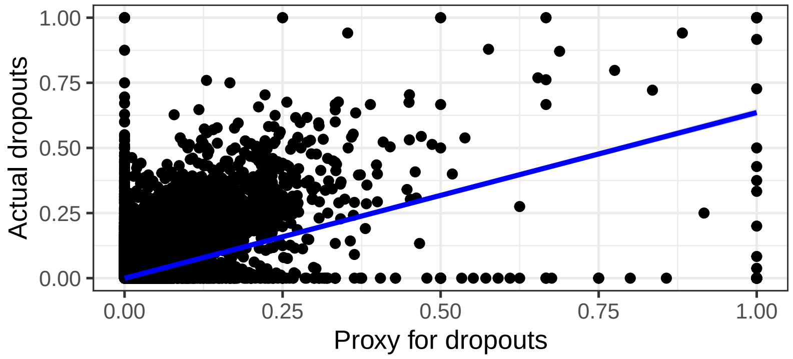

Notes: The figure plots classroom-level dropouts according to administrative data (on the vertical axis) and according to our proxy (on the horizontal axis), for Q4-2019 school year. Administrative dropouts = 1 for students enrolled at a State public school in 2019 but not in 2020, and 0 otherwise. High dropout risk = 1 for students without math and Portuguese grades on record at Q4, and 0 otherwise. The regression line is estimated through OLS.

A.2 Odd ratios

An alternative way to gauge the predictive power of our proxy is to compute odds ratios, comparing the probability of participation in school activities of students who had missing scorecard grades in the previous year to that of other students. This section does that for actual re-enrollment in 2020, to validate the proxy with pre-pandemic data, as well as for zero attendance and missing scorecard grades in Q1/2021, to validate it in the context of COVID-19.

Let y_{igt} be a measure of student dropouts for student i, enrolled in grade g at year t, and let $\tilde{y}_{igt}$ be its corresponding proxy. To simplify notation, let $p = \frac{\sum_i \boldsymbol{I}[y_{igt}=1]}{\sum_i \boldsymbol{I}[\tilde{y}_{igt}=1]}$ and $q = \frac{\sum_i \boldsymbol{I}[y_{igt}=1]}{\sum_i \boldsymbol{I}[\tilde{y}_{igt}=0]}$, where $\boldsymbol{I}[.]$ is the indicator function, define conditional probabilities of actual dropout, respectively when proxy dropouts $= 1$ or $= 0$. For each measure of student dropouts, we compute odds ratios as:

$$OR = \frac{p(1-q)}{q(1-p)} \tag{1}$$

Simply put, we calculate how much more likely students with missing scorecard grades are not to re-enroll in 2020 or not to participate in school activities over Q1/2021, relative to other students. We can compute standard-errors for the log of these odd ratios using the following analytical formula:

$$SE\left(log(OR)\right) = \sqrt{\frac{1}{p} + \frac{1}{(1-p)} + \frac{1}{q} + \frac{1}{(1-q)}}, \tag{2}$$

and using the Delta method approximation, we can write the standard-errors for the odd ratios itself as (*1*):

$$SE(OR) = \sqrt{\frac{1}{p} + \frac{1}{(1-p)} + \frac{1}{q} + \frac{1}{(1-q)}} * \left(\frac{p(1-q)}{q(1-p)}\right) \tag{3}$$

Table S.A.1 reports the results. Column (1) shows that students with missing math and Portuguese scorecard grades in 2019 are seven times more likely not to be enrolled in the following year than other students, validating the proxy prior to the pandemic. Since the connection between our proxy and actual dropouts might have changed during such exceptional times – concretely, many students might have failed to hand in homework and take exams during the pandemic due to atypical circumstances, from connectivity constraints to concerns about being exposed to COVID-19 – columns (2) and (3) estimate odds ratios for indicators of school participation in 2021, when in-person classes had been authorized to return by all municipalities of São Paulo State. We focus on whether students engaged in *any* academic activity during this school quarter: attending classes or taking exams that would qualify for scorecard grades in Q1/2021, across all school subjects. If a student missed all classes across all subjects during the whole school quarter, this is essentially equivalent to not being enrolled in school. If our proxy still predicts intended non-enrollment in 2020, we would expect that students at high dropout risk during that year are less likely to participate in any academic activity in 2021. The table shows that students with missing scorecard grades in Q4/2020 were 8.6 times more likely not to have attended a single class (column 2) and 9.7 times more likely not to have taken a single exam (column 3) in Q1/2021. We reject the null hypothesis that each odds ratio is equal to one at the 1% level. As such, we conclude that this proxy remains a strong predictor of student dropouts even throughout the pandemic.

Table S.A.1: Odds ratios for different measures of student engagement relative to missing scorecard grades

	(1) Not enrollment in 2020	(2) No classes attended in Q1 2021	(3) No tests taken in Q1 2021
Odds Ratio	7.21	8.65	9.78
	(0.001)	(0.001)	(0.001)
N	1,969,552	1,606,909	

Notes: The table shows odds ratios computed using equation (1) for no re-enrollment in 2020 (column 1), zero attendance across all classes in 2021's first school quarter (column 2), and no scorecard grades in 2021's first school quarter (column 3), contrasting students with missing scorecard grades for Portuguese and Math in Q4/2020 to all other students. All columns restrict attention to 6th-11th grades. Analytical approximations for the standard errors of the logarithm of odds ratios in parentheses, computed using equation (2). P-values computed from two-sided t-tests that each odds ratio is equal to one.

A.3 Correcting for measurement error

Last, we can assess the sensitivity of our results to directly correcting for measurement error from not directly observing actual dropouts. Ideally, we would like to estimate the following difference-in-differences regression:

$$y^T_{itg} = x_{igt}\beta + \epsilon_{igt}, \tag{4}$$

where y^T_{igt} is an indicator variable of whether student i enrolled in grade g at year t has dropped out of schools; x_{igt} is a vector of covariates including an indicator variable for the 2020 school year, an indicator variable for Q4, an interaction between the two, and grade fixed-effects; ϵ_{igt} is an error term. We are interested in the interaction variable that recovers the difference-in-differences coefficient, shown in columns (3)-(5) of Table 1.

The regression above is, however, unfeasible, because we do not observe y. How replacing it with the proxy $\tilde{y}$ changes results depends on the connection between the y and $\tilde{y}$. Let:

$$y_{igt} = \tilde{y}_{igt} + v_{igt}, \tag{5}$$

where v_{igt} is a proxy classification error. If measurement error were classical (random, distributed according to a continuous zero-mean distribution), using a proxy would not bias our estimates, but only decrease their precision (*2*). However, since in our application the latent variable and the proxy are both binary, classification error cannot be classical (*3*). Instead:

$$v_{igt} = y_{igt} - \tilde{y}_{igt} = \begin{cases} 1, \text{for false negatives} \\ 0, \text{for accurate predictions} \\ -1, \text{for false positives} \end{cases} \tag{6}$$

Let the probabilities of false positives and false negatives be:

$$P(\tilde{y}_{igt} = 1 | y_{igt} = 0) = \alpha_0 \tag{7}$$

$$P(\tilde{y}_{igt} = 0 | y_{igt} = 1) = \alpha_1 \tag{8}$$

These probabilities are tightly connected to the odds ratios estimated in the previous section. Since the proxy predicts the dependent variable, it is likely correlated with the explanatory variables (X) included in the regression. Thus, the use of the proxy creates a correlation between covariates and measurement error, which affects estimates of β.

Let δ be the difference between the desired coefficient (β) and the feasible estimate ($\tilde{\beta}$). Then:

$$\mathbb{E}(\delta) = \mathbb{E}(\hat{\hat{\beta}}) - \beta \tag{9}$$

Since the error term only assumes three values, it is straightforward to derive a close formula for the difference between feasible and desirable coefficients:

$$\begin{aligned}\mathbb{E}(\delta) = N(XX')\Big[&Pr(y_{igt} = 1, \tilde{y}_{igt} = 0)E[X|y_{igt} = 1, \tilde{y}_{igt} = 0]- \\ &-Pr(y_{igt} = 0, \tilde{y}_{igt} = 1)E[X|y_{igt} = 0, \tilde{y}_{igt} = 1]\Big]\end{aligned} \tag{10}$$

For the particular case that measurement error is constant across X, the expression above simplifies to:

$$\mathbb{E}(\delta) = (\alpha_0 + \alpha_1)\beta \tag{11}$$

$\mathbb{E}(\delta)$ captures the expected difference between our estimates ($\hat{\hat{\beta}}$) and the coefficient we would obtain if we observe actual dropouts ($\hat{\beta}$). For instance, the coefficient reported in Panel A of Table 1 (columns 3-5) is 0.0621. If measurement error were constant across X, we could use equation (10) to correct for it by computing:

$$\hat{\beta} = \frac{0.0621}{1 - \alpha_0 - \alpha_1} \tag{12}$$

Of course, we do not directly observe false positive or false negative rates (α_0 and α_1) that relate our proxy to actual dropouts in 2021 (otherwise, there would be no need to use a proxy). However, we do observe $\hat{\alpha}_0$ and $\hat{\alpha}_1$, the false positive and false negative rates connecting proxy and actual dropouts in 2020. As such, we can correct our estimates for measurement error by plugging in false positive and negative rates for 2020 in the equation above. Because the connection between proxy and actual dropout might have changed during the pandemic, we alternatively compute false positive and negative rates using the measures of school participation in 2021 discussed in Section A2 of Supplementary Materials.

Supplementary Table A.2 reports estimates for treatment effects on proxy dropouts corrected for measurement error according to the procedure above. Column (1) assumes that measurement error is constant across X, and corrects coefficients using equation (10). Column (2) allows measurement error to be correlated with covariates – in particular, it allows it to vary by grade or school quarter –, correcting coefficients using equation (9). Panel A estimates false positive and false negative rates based on actual dropouts in 2020; Panel B, based on zero attendance in Q1 2021; and Panel C, based on no scorecard grades in Q1/2021. All corrected estimates are very close to the ones estimated in Table 1; if anything, corrected estimates are 4-12% higher than coefficients reported in Table 1.

Table S.A.2: Effects of remote learning on dropout rates correcting for measurement error

	(Q4 2020-Q1 2020)-(Q4 2019-Q1 2019) (1)	(2)
**Panel A: Actual Dropouts in 2020**		
Remote learning	0.070	0.068
	(0.0002)	(0.0002)
**Panel B: Zero attendance in Q1/2021**		
Remote learning	0.068	0.067
	(0.0002)	(0.0002)
**Panel C: Missing all scorecard grades in Q1/2021**		
Remote learning	0.065	0.065
	(0.0002)	(0.0002)
N	8,543,856	
Grade fixed-effects	yes	yes
Matching	no	no

Notes: The table displays treatment effects of remote learning on proxy dropouts correcting for measurement error. In all columns, we estimate differences-in-differences comparing variation in outcomes between Q1- and Q4-2020 to that between Q1- and Q4-2019. The dependent variable is high dropout risk (= 1 if the student had no math or Portuguese grades on record for that school quarter, and 0 otherwise). We correct for classification error using equation (10) in column (1) and equation (9) in column (2). False positive and false negative rates are estimated using actual dropouts in 2020 in Panel A, zero attendance in Q1/2021 in Panel B, and missing all scorecard grades in Q1/2021 in Panel C. All columns include grade fixed-effects and an indicator variable equal to 1 for municipalities that authorized schools to reopen from Sep-2020 onward, and 0 otherwise (allowing its effects to vary at Q4). All columns are OLS regressions, with bootstrapped standard errors. P-values computed from two-sided t-tests that each coefficient is equal to zero.

All in all, we conclude that our proxy of student dropouts allows us to accurately capture the effects of remote learning on actual dropouts despite classification error.

B. Descriptive statistics

Supplementary Figure B.1 showcases aggregate trends in educational outcomes across schools quarters for 2018-2020, separately for middle- and high-school students.

Figure S.B.1: Trends in dropout risk and standardized test scores

Panel A: Middle-school students

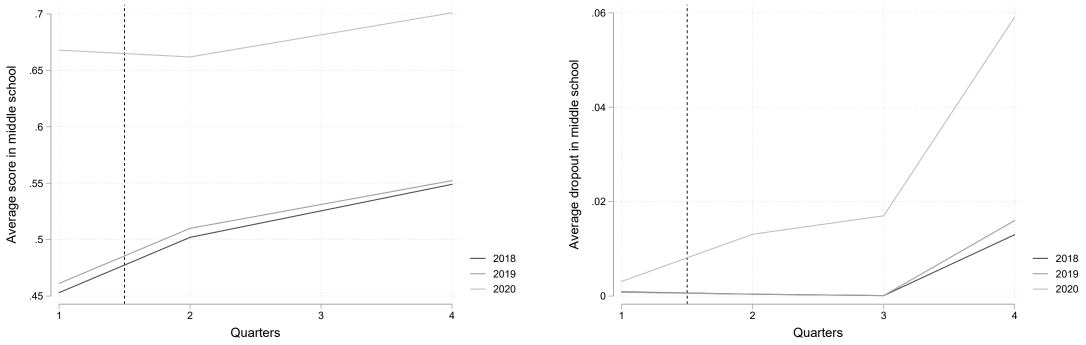

Panel B: High-school students

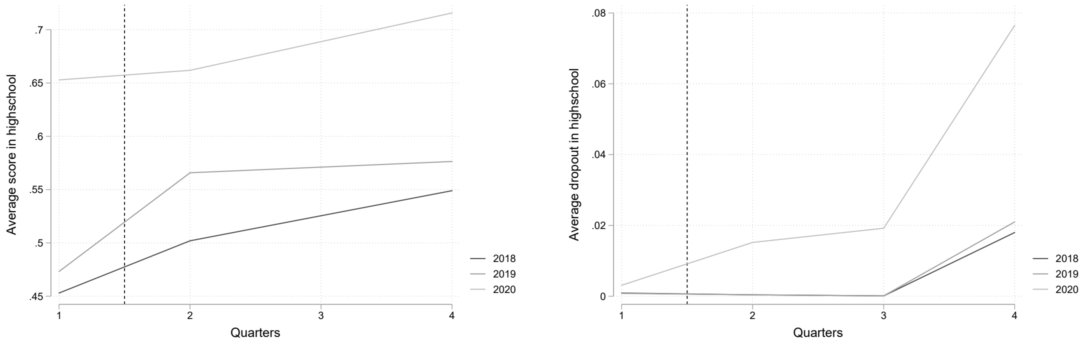

Notes: The figure showcases trends in high dropout risk and standardized test scores across school quarters for 2018, 2019 and 2020, pooling data for all middle-school students (Panel A) and all high-school students (Panel B). High dropout risk = 1 if the student had no math or Portuguese grades on record for that school quarter, and 0 otherwise. Standardized test scores from quarterly standardized tests ($AAPs$), averaging math and Portuguese scores for that school quarter.

C. Additional details on standardized tests

C.1 Description of the standardized evaluations

The São Paulo State Secretariat (SEDUC) conducts standardized tests (Avaliações de Aprendizagem em Processo, AAPs) with the aim of evaluating students' quarterly progress in Math and Portuguese. Participation in these tests is *not* mandatory, and students who do not participate or those with low scores are not penalized in any way. Having said that, SEDUC strongly incentivizes participation. Schools are required to print materials promoting each test, and to recurrently remind and motivate students to take part in the exam. Such engagement ensured a participation rate of no less than 80% in each and every test throughout 2019 and 2020 – even in those conducted remotely over the course of the pandemic.

The evaluation consists of one math and one Portuguese exam each school quarter. The exams started off as a pilot in 2011, and remained in the same format between 2015 and 2019. Each year, a group of public school teachers is designated to prepare questions for the exams following guidelines on the topics and difficulty level. This is meant to make test scores comparable across years (*4*). AAPs have been found to contribute to the teaching of Portuguese and to the identification of learning setbacks in specific subjects (*5*).

In 2020, all exams were applied online (alternatively, students without access to connectivity could fetch printouts at the school gate, and return them the same way). Students had 48 hours to complete the exam. Questions for the exam were prepared the same way as in previous years, except that in 2020 the guidelines for the school curriculum were simplified as soon as it was clear that in-person classes would have to be suspended, to account for the fact that remote learning would not be able to cover as much (*6*). Exams were applied consistently throughout all schools quarters of 2020, which enables the within-year comparisons we pursue in the main text.

One important issue is potential cheating in the remote application of the standardized tests. While the Education Secretary had no enforceable mechanism to prevent cheating in remote exams, as discussed above, students had no discernible benefits (costs) from scoring high (low) in the AAPs. Most importantly, as long as cheating is not differential between Q1 and Q2-Q4, it does not affect the comparisons of interests in the main text. Moreover, Appendix C.2 shows that while the distribution of GPA (a key determinant of whether the student graduates or advances to the next grade) considerably changed in 2020 relative to previous years – with significant bunching on minimum passing grades –, the same did *not* happen with the distribution of AAPs' scores, which displays no evidence of bunching neither in previous years nor during the pandemic.

C.2 Comparability between 2019 and 2020 test scores

A potential concern with our econometric results is that standardized test scores in 2020 might not be comparable to those in 2019. In the main text, we discussed that they are not directly comparable in one key dimension: in 2020, exams were taken remotely. That difference is consequential – average standardized test scores are substantially higher in 2020, relative to 2019 (as Appendix B shows). We account for that difference in our empirical strategy by comparing *changes* in test scores within 2020 (under remote exams) to those within 2019 (under in-person exams). While there were other changes in standardized tests between 2019 and 2020 – in particular, the simplified curriculum recommended for Brazilian schools during the pandemic(*6*) was reflected in 2020 standardized tests(*7*) –, most importantly, such changes were *not* differential across school quarters: the Q1-2020

AAP already reflected the simplified curriculum, benefiting from re-planning efforts that happened early on, as the state of the pandemic worsened in the country.

Having said that, if standardized tests were graded *disproportionately* favourably in Q1-2020, as classes were transitioning from in-person to remote, our strategy would still overestimate learning losses under remote classes. This Appendix provides evidence against this hypothesis. While we do observe strategic grading in Q1-2020 with respect to student GPA, we do *not* find similar evidence with respect to standardized tests scores.

Supplementary Figure C.1 showcases that GPA grading changed considerably between Q1-2019 and Q1-2020. In 2019, the distribution of grades was close to a normal distribution. In 2020, in turn, we observe considerable bunching around the minimum passing grade. Besides minimum attendance, GPA is the key variable determining grade progression. Since grading for regular exams is decentralized at the teacher level, teachers might have felt like they had a mandate to try to prevent students from falling through the cracks in such a difficult time. The State later changed the grade progression rules, preventing grade repetition for almost every student in 2020 – rendering such manipulation ultimately unimportant, although revealing of teachers' strategic grading behavior.

Figure S.C.1 GPA distribution in Q1-2019 and Q1-2020

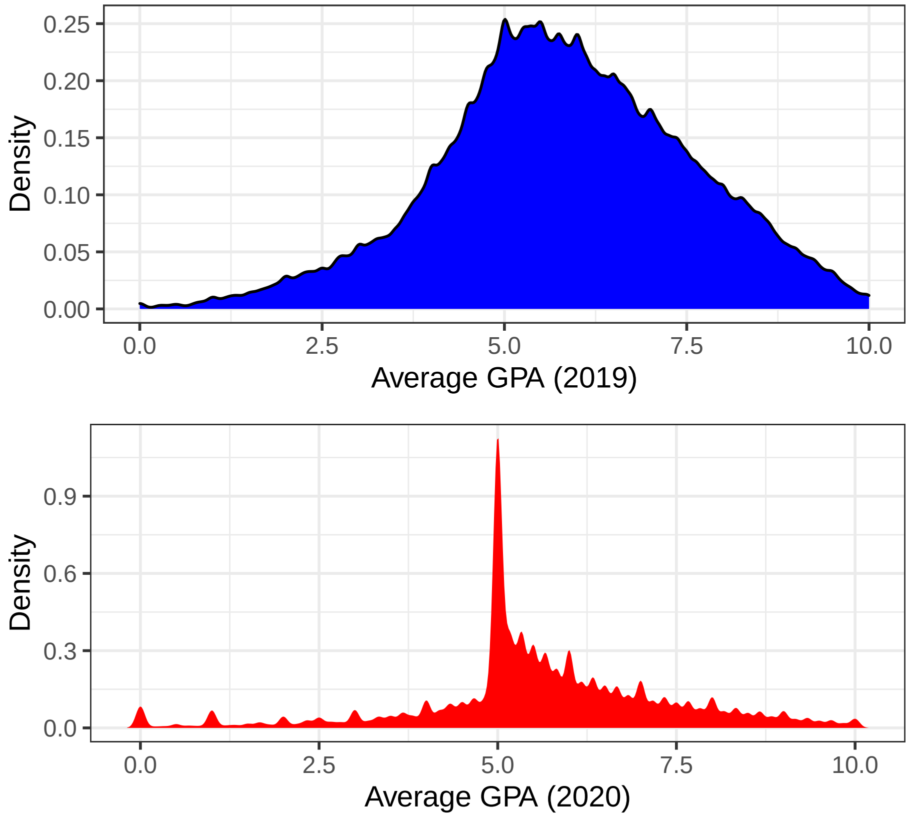

Notes: The figure shows the GPA distribution for all students in Q1-2019 and Q1-2020. Math and Portuguese GPA were averaged at the student-quarter level.

For standardized tests, however, such incentives for strategic manipulation were absent from the get-go. Such tests are optional, with no bearing on students' academic prospects, and they are not graded by the teachers, but rather by external graders.

In fact, Supplementary Figure C.2 shows that, unlike GPA, the distribution of standardized tests scores in Q1-2019 displays no evidence of bunching relative to that of Q1-2020.

Figure S.C.2 Distribution of standardized test scores in Q1-2019 and Q1-2020

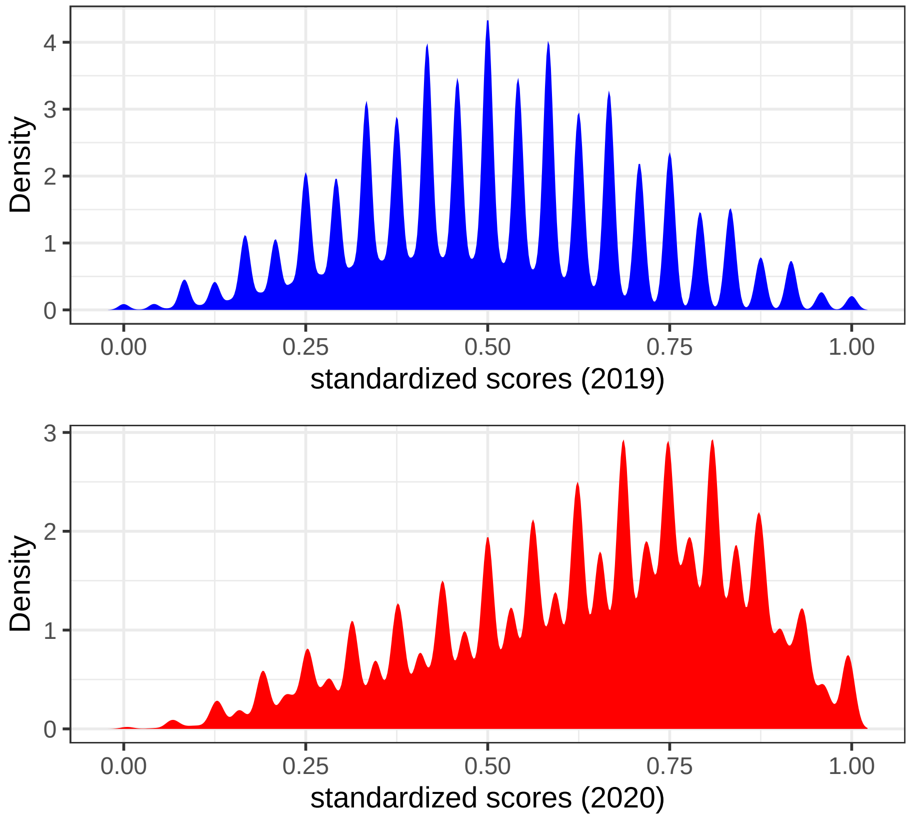

Notes: The figure shows the distribution of standardized scores for all students in Q1-2019 and Q1-2020. Math and Portuguese GPA were averaged at the student-quarter level.

The non-smoothness in the distributions (both in 2019 and 2020) reflect the fact that we only have access to standardized test scores rounded to the closest integer. While the 2020 distribution clearly has more mass on higher scores, Appendix D shows that average standardized test scores are higher throughout 2020, relative to 2020. In fact, such scores do increase between Q1- and Q4-2020; just not as much as in the counterfactual, under in-person classes.

D. Heterogeneous treatment effects by characteristics

Supplementary Figures D.1 and D.2 showcase average standardized test scores and dropout risk, respectively, by school quarter of 2019 and 2020 and by selected student and school characteristics.

Figure S.D.1: Average standardized test scores, by school quarter and sub-group

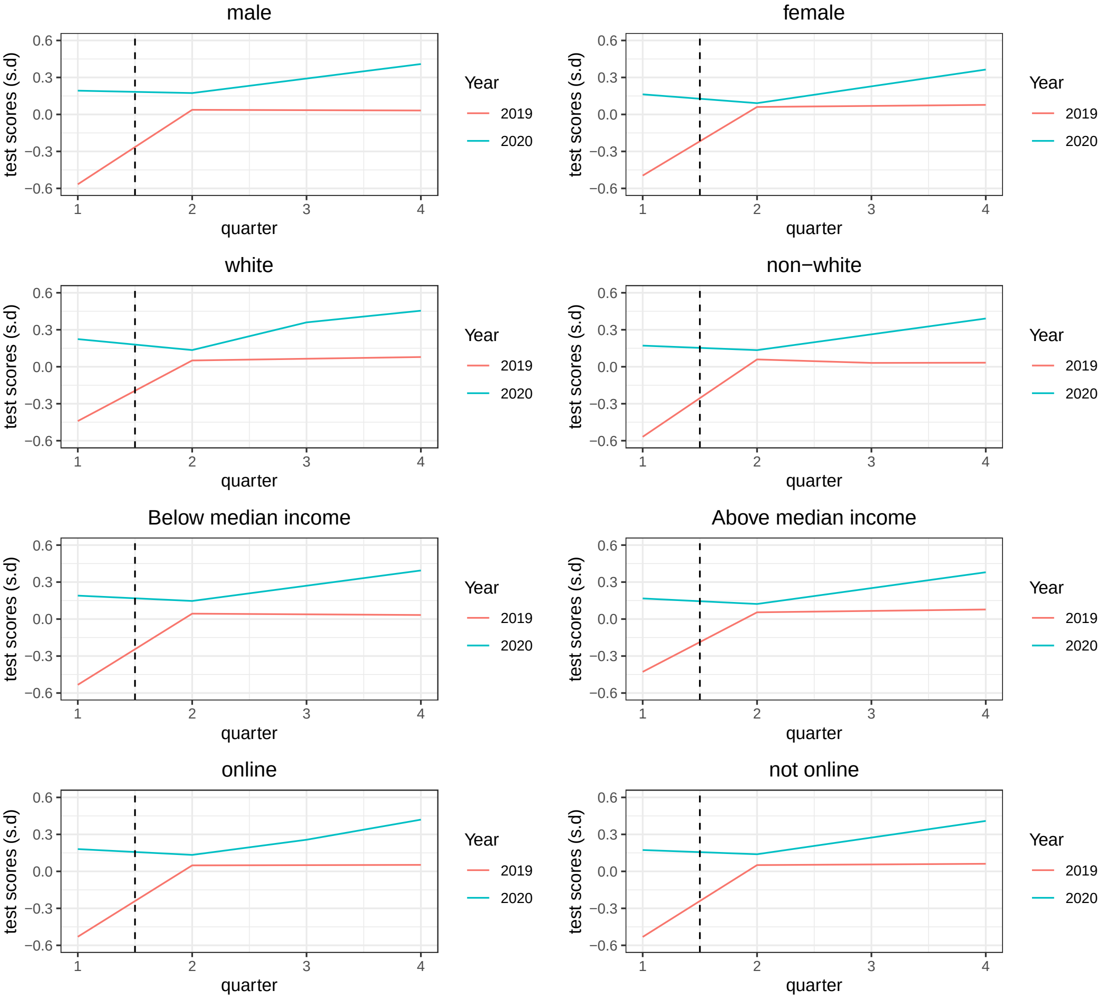

Notes: Quarterly average standardized test scores, computed by restricting observations to each quarter and sub-group. Sub-groups included are (1) student gender (male or female); (2) student race (white, also comprising students whose declared race is yellow or Asian, or non-white; i.e., black, brown and indigenous students); (3) neighborhood income (schools located in below- or above-median per capita income neighborhoods, according to the 2010 Census); and (4) prior engagement in online activities (schools with or without online academic activities prior to the pandemic, according to the 2019 Educational Census).

Figure S.D.2: Average high dropout risk, by school quarter and sub-group

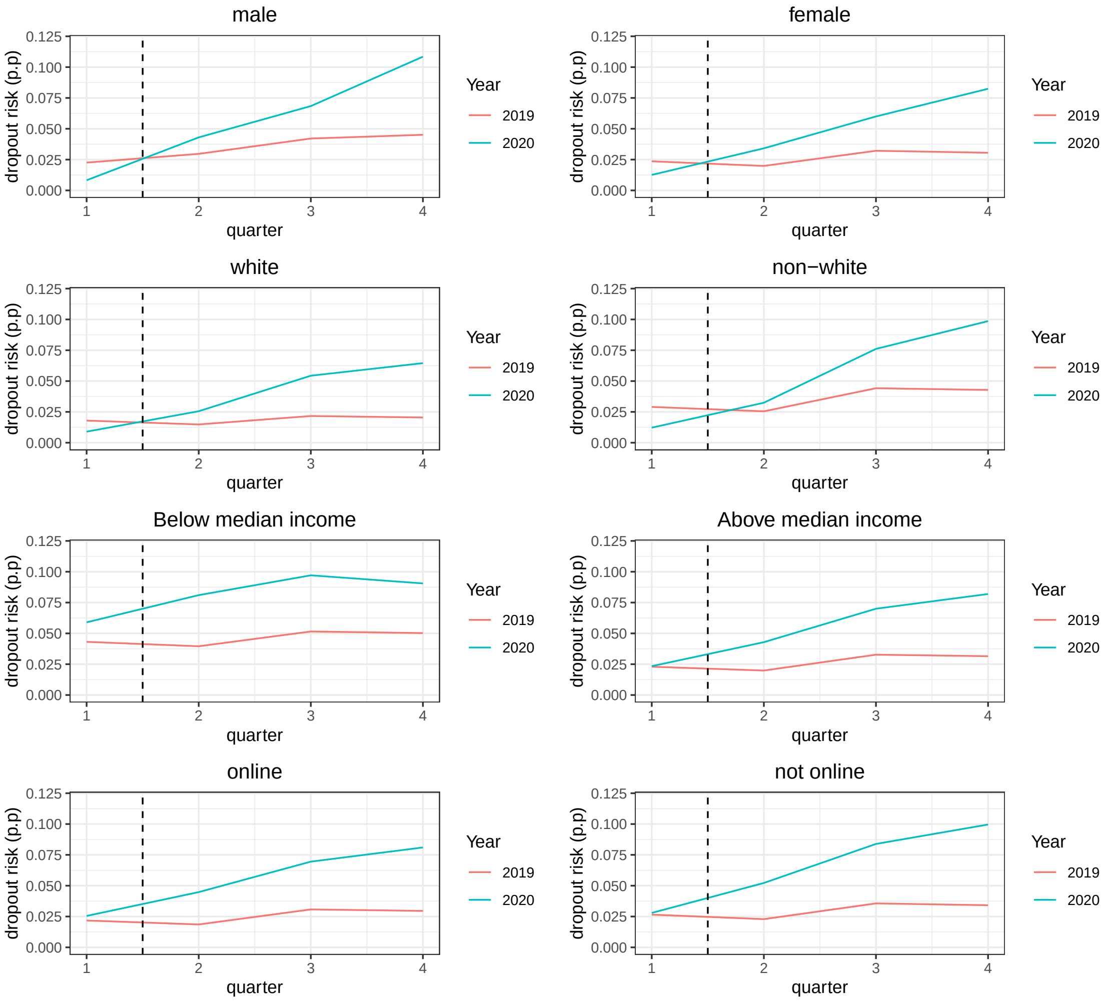

Notes: Quarterly average high dropout risk, computed by restricting observations to each quarter and sub-group. Sub-groups included are (1) student gender (male or female); (2) student race (white, also comprising students whose declared race is yellow or Asian, or non-white; i.e., black, brown and indigenous students); (3) neighborhood income (schools located in below- or above-median per capita income neighborhoods, according to the 2010 Census); and (4) prior engagement in online activities (schools with or without online academic activities prior to the pandemic, according to the 2019 Educational Census).

Supplementary Table D.1 reports differences-in-differences estimates of the impacts of remote learning for each sub-group.

Table S.D.1: Heterogeneous treatment effects of remote learning on test scores and dropout risk

Group	Treatment effects	
	Std. test scores	High dropout risk
Male	-0.321	0.060
	(0.0006)	(0.0003)
Female	-0.321	0.071
	(0.0006)	(0.0003)
p-value difference	0.54	<0.001
White	-0.289	0.068
	(0.0006)	(0.0003)
Non-white	-0.344	0.081
p-value difference	<0.001	<0.001
	(0.0009)	(0.0005)
High income	-0.291	0.054
	(0.0006)	(0.0003)
Low income	-0.420	0.091
	(0.0006)	(0.0003)
p-value difference	<0.001	<0.001
Online	-0.271	0.053
	(0.0006)	(0.0004)
Not online	-0.431	0.090
	(0.0006)	(0.0003)
p-value difference	<0.001	<0.001

Notes: The Table displays treatment effects of remote learning on educational outcomes, based on regressions following the specification in Column (5) of Table 1, only restricting observations to each sub-group. Sub-groups included are (1) student gender (male or female); (2) student race (white, also comprising students whose declared race is yellow or Asian, or non-white; i.e., black, brown and indigenous students); (3) neighborhood income (schools located in below- or above-median per capita income neighborhoods, according to the 2010 Census); and (4) prior engagement in online activities (schools with or without online academic activities prior to the pandemic, according to the 2019 Educational Census). For each pair of groups, we present the p-value for the difference between estimated coefficients for these groups. Standardized test scores from quarterly standardized tests (*AAPs*), averaging math and Portuguese scores for that school quarter. High dropout risk = 1 if the student had no math or Portuguese grades on record for that school quarter, and 0 otherwise. All estimates absorb grade fixed-effects, parse out the effects of school reopening, control for a third-degree polynomial of the propensity score, and re-weight observations by the inverse of their propensity score. All columns are OLS regressions, with standard errors clustered at the school level.P-values computed from two-sided t-tests that each coefficient is equal to zero.

While Supplementary Figure D.1 suggests that girls – whose average test scores were higher than those of boys by Q4/2019, but lower by Q4/2020 – were hurt disproportionately by remote learning, Supplementary Table D.1 shows that this is actually *not* the case, at least when it comes to test scores. That false impression stems from naive comparisons of test scores coming from different assessment modes; in effect, the differences-in-differences estimate documents that remote learning hurt boys' and girls' test scores by exactly the same extent. When it comes to dropout risk, however, girls were really hurt to

a greater extent (a nearly 18% higher effect size, significant at the 1% level). Supplementary Table D.1 also shows that, relative to white students, non-whites experienced 19% larger losses in test scores due to remote learning ($p<0.001$) and a 19% larger increase in dropout risk ($p<0.001$). Strikingly, learning losses due to remote learning were much more dramatic in low-income schools: relative to schools located in above-median per capita income neighborhoods, the former experienced a 44% larger impacts on standardized test scores ($p<0.001$) and a 69% larger increase in dropout risk ($p<0.001$). Similarly, schools without online academic activities prior to the pandemic experienced 59% larger impacts on standardized test scores ($p<0.001$) and a 70% larger increase in dropout risk ($p<0.001$).

E. Additional results

Columns (1-2) in Supplementary Table E.1 report mean values for student characteristics, separately for those who took any standardized test in 2019 (column 1) and in 2020 (column 2). While most differences seem small, one can see that, in 2020, not only attendance and grades were higher among the sample with standardized test scores, but also, their characteristics indicate that they are indeed positively selected: there is a higher share of white students among test-takers, who also tend to come from higher-income schools and more likely to have offered online activities prior to the pandemic. Differences across the two samples are indeed highly statistically significant (p-value of an F-test of joint significance < 0.001). Next, columns (3-4) report characteristics of the sample of 2020 after applying matching procedures: column (3) displays those using inverse probability weights, and column (4), controlling for the propensity score. Sample means under both procedures approximate those of the 2019 sample to a much better extent; in particular, one can see that the proportion of white students, the average per capita income of the school neighborhood and the share of test-takers from schools with online activities prior to the pandemic are (nearly) identical across matched samples. As a result, we no longer reject the hypothesis that the samples are balanced at conventional significance levels in each case (p=0.52 and 0.31, respectively).

Table S.E.1: Student and school characteristics among those who took standardized tests in 2019 and 2020, with and without matching

	2019 sample	2020 sample	2020 sample with inverse probability weighting	2020 sample controlling for propensity scores
Attendance	0.89	0.95	0.91	0.91
Scorecard grades	6.21	6.39	6.20	6.21
Male	0.48	0.51	0.51	0.51
White	0.55	0.59	0.55	0.56
Per capita income (R$)	913.00	920.38	912.93	913.08
Prior online activities	0.72	0.74	0.72	0.72
p-value(F-test)		0.00	0.52	0.31

Notes: The table displays average student and school characteristics among the sub-sample who took any standardized tests in 2019 (column 1) and in 2020 (column 2). Column (3) re-weights observations in the 2020 sample using the inverse of the propensity scores. Column (4) residualizes student and school characteristics controlling for a cubic polynomial of the propensity score. P-values for the two-sided F-test that all means are jointly equal to those in the 2019 sample.

Supplementary Table E.2 presents the marginal probability changes associated with selection into non-null standardized test scores in Q4-2020, relative to Q4-2019. For illustration purposes, the table estimates across all grades, and only displays selected variables); in turn, the propensity scores that we use for both matching and re-weighting observations in the main text are estimated separately for each grade and quarter.

As the table shows, the profile of students who take the standardized test in the last quarters of 2019 and 2020 changes significantly between the two years. In particular, girls, non-white, and under-performing students are under-represented in 2020, as those in schools located in poorer neighborhoods – highlighting that matching and re-weighting are

critical for unbiased estimates, especially in face of heterogeneity of treatment effects.

Table S.E.2: Selection into Q4 standardized test (across all grades)

	Marginal probability change
White x 2020	0.051
	(0.001)
Male x 2020	0.005
	(0.001)
Scorecard grade x 2020 (10 scale)	0.045
	(0.001)
Scorecard frequency x 2020 (100 scale)	-0.001
	(0.001)
Income x 2020 (thousand R$)	0.011
	(0.001)

Notes: The table shows marginal probability changes associated with selected variables in a Probit model. The dependent variable is a dummy for taking at least one standardized test over the course of the school year. Additional variables not shown are indicator variables for high school (and its interaction with the 2020 indicator), whites, and males; school attendance; scorecard grades; and school neighborhood's per capita income. Standard errors clustered at the municipality level in parenthesis. P-values computed from two-sided t-tests that each coefficient is equal to zero.

In Supplementary Table E.3, we present a slight variation of the main results shown in Table 1, estimating the differences-in-differences model with 2018 instead of 2019 as the counterfactual year. Results are very robust to that alternative definition.

Table S.E.3: Effects of remote learning on dropout risk and test scores with alternative baseline period

	(Q4 2020-Q1 2020)-(Q4 2018-Q1 2018)		
	(1)	(2)	(3)
**Panel A**: High dropout risk			
Remote learning	0.0655	0.0655	0.0655
	(0.0002)	(0.0002)	(0.0002)
Mean 2018 Q4	0.016	0.016	0.016
N		8,312,220	
**Panel B**: Standardized test scores			
Remote learning	-0.304	-0.291	-0.310
	(0.0001)	(0.0001)	(0.0001)
In-person learning equivalent	0.44	0.44	0.44
N		7,001,012	
Grade fixed-effects	yes	yes	yes
Matching	no	yes	yes
Inverse probability weighting	no	no	yes

Notes: The table displays treatment effects of remote learning on educational outcomes. In all Columns, we estimate differences-in-differencess comparing variation in outcomes between Q1- and Q4-2020 to that

between Q1- and Q4-2018. In Panel A, the dependent variable is high dropout risk (= 1 if the student had no math or Portuguese grades on record for that school quarter, and 0 otherwise). In Panel B, the dependent variable is scores from quarterly standardized tests (*AAPs*), averaging math and Portuguese scores for that school quarter. All columns include grade fixed-effects and an indicator variable equal to 1 for municipalities that authorized schools to reopen from Sep-2020 onward, and 0 otherwise (allowing its effects to vary at Q4). In Columns (1) and (2), we control for the propensity score of selection into exams with third-degree polynomial. In Column (3), we also re-weight observations by the inverse of their propensity score. All columns are OLS regressions, with standard errors clustered at the school level. P-values computed from two-sided t-tests that each coefficient is equal to zero.

In Supplementary Table E.4, we show treatment effects separately for Portuguese and math scores. Since we do not have data on grades separately for each subject for Q4/2020, the table estimates treatment effects of remote learning separately for math and Portuguese grades based off differences between Q1 and Q2-Q3 within 2020 relative to those within 2019.

Table S.E.4: Effects of remote learning on test scores, separately for Portuguese and math

	(Q3 2020-Q1 2020)-(Q3 2019-Q1 2019)		
	(1)	(2)	(3)
**Panel A**: Portuguese test scores			
Remote learning	-0.255	-0.265	-0.267
	(0.0002)	(0.0002)	(0.0002)
In-person learning equivalent	0.44	0.44	0.44
N		7,131,922	
**Panel B**: Math test scores			
Remote learning	-0.361	-0.342	-0.355
	(0.0002)	(0.0002)	(0.0002)
In-person learning equivalent	0.44	0.44	0.44
N		7,131,922	
Grade fixed-effects	yes	yes	yes
Matching	no	yes	yes
Inverse probability weighting	no	no	yes

Notes: The table displays treatment effects of remote learning on educational outcomes. In all Columns, we estimate differences-in-differencess comparing variation in outcomes between Q1- and Q3-2020 to that between Q1- and Q3-2019. In Panel A, the dependent variable is math test scores and, in Panel B, Portuguese test scores. All columns include grade fixed-effects and an indicator variable equal to 1 for municipalities that authorized schools to reopen from Sep-2020 onward, and 0 otherwise (allowing its effects to vary at Q4). In Columns (1) and (2), we control for the propensity score of selection into exams with third-degree polynomial. In Column (3), we also re-weight observations by the inverse of their propensity score. All columns are OLS regressions, with standard errors clustered at the school level. P-values computed from two-sided t-tests that each coefficient is equal to zero.

Supplementary Table E.4 documents that learning losses due to remote learning are massive for both subjects, but especially so for math. While students learned only 40% of they would have learned under in-person classes in Portuguese, that figure was only 20% in math classes.

In Supplementary Table E.5, we estimate a differences-in-differences model, similar to

the one shown in Table 2, but using a continuous variable for the number of weeks each municipality reopened schools.

Table S.E.5: ITT effects of resuming in-person school activities on dropout risk and test scores with continuous treatment

	(1) Attendance	(2) Std. test scores	(3) Dropout risk
**Panel A**: Diff-in-diff: Middle school			
In-person activities	0.001	0.002	0.0002
	(0.001)	(0.001)	(0.001)
**Panel B**: Diff-in-diff: High school			
In-person activities	0.0001	0.005	0.0003
	(0.001)	(0.0002)	(0.0002)
**Panel C**: Triple differences			
In-person activities	-0.00001	0.003	0.0001
	(0.002)	(0.0001)	(0.0001)
Grade fixed-effects	yes	yes	yes
Matching	yes	yes	yes
N	3,701,482	2,624,943	3,701,482

Notes: The table displays intention-to-treat (ITT) treatment effects of the number of weeks municipalities reopened schools for in-person school activities on student attendance (Column 1), standardized test scores (Column 2) and high dropout risk (Column 3). Quarterly data on attendance reflects online or in-person attendance and/or assignment completion (handed in online or in-person) over each quarter (in p.p.), averaged across math and Portuguese classes; standardized test scores from quarterly standardized tests (*AAPs*), averaging math and Portuguese scores for that school quarter; and high dropout risk = 1 if the student had no math or Portuguese grades on record for that school quarter, and 0 otherwise. In Panels A and B, we estimate treatment effects through differences-in-differencess, contrasting variation in outcomes between Q1- and Q4-2020 within municipalities that authorized schools to reopen and those that did not. Panel A restricts attention to middle-school students, and Panel B, to high-school students. Panel C estimates treatment effects through a triple-differences estimator, which contrasts the differences-in-differences estimates for middle- and high-school students (for whom in-person classes could return within municipalities that authorized schools to reopen in Q4-2020). Column (2) controls for a third-degree polynomial of propensity scores, and re-weights observations by the inverse of their propensity score. All columns are OLS regressions, with standard errors clustered at the municipality level. P-values computed from two-sided t-tests that each coefficient is equal to zero.

In Supplementary Table E.6, we replicate the results in Table 2, but implement the matching at the municipality-level, instead of the student-level.

Table S.E.6: ITT effects of resuming in-person school activities on dropout risk and test scores with municipality-matching

	(1) Attendance	(2) Std. test scores	(3) Dropout risk
**Panel A**: Diff-in-diff: Middle school			
In-person activities	0.009	0.001	0.001
	(0.001)	(0.001)	(0.001)
**Panel B**: Diff-in-diff: High school			
In-person classes	0.008	0.021	0.002
	(0.001)	(0.0001)	(0.002)
Grade fixed-effects	yes	yes	yes
Municipality matching	yes	yes	yes
N	3,701,482	2,624,943	3,701,482

Notes: The table displays intention-to-treat (ITT) treatment effects of resuming in-person school activities on student attendance (Column 1), standardized test scores (Column 2) and high dropout risk (Column 3). Quarterly data on attendance reflects online or in-person attendance and/or assignment completion (handed in online or in-person) over each quarter (in p.p.), averaged across math and Portuguese classes; standardized test scores from quarterly standardized tests (*AAPs*), averaging math and Portuguese scores for that school quarter; and high dropout risk = 1 if the student had no math or Portuguese grades on record for that school quarter, and 0 otherwise. we estimate treatment effects through differences-in-differences, contrasting variation in outcomes between Q1- and Q4-2020 within municipalities that authorized schools to reopen and those that did not. Panel A restricts attention to middle-school students, and Panel B, to high-school students. For all columns, we match municipalities using variables in Supplementary Table G.1. All estimates are OLS regressions, with standard errors clustered at the municipality level. P-values computed from two-sided t-tests that each coefficient is equal to zero.

Supplementary Table E.7 reports sensitivity tests for selection effects. For this analyses, we track students who took standardized tests in Q4/2019 and in Q4/2020. In Panel A, we estimate how standardized test scores differ for those students, relative to other students, to document the extent of selection. In Panel B, we re-estimate treatment effects of remote learning on standardized test scores using differences-in-differences with a balanced panel, restricting attention to students who took all exams.

Panel A shows that standardized test scores in 2019 were 0.09 standard-deviations higher in the selected sample relative to other students (significant at the 1% level), confirming that they are indeed positively selected. In turn, Panel B shows that, even among this highly selected sample, the effects of remote learning were substantially negative. We document that learning losses relative to in-person classes were of the order of 0.225 s.d. (significant at the 1% level) – over 70% of the coefficient reported in Table 1.

Table S.E.7: Sensitivity tests for selection effects

**Panel A**: Standardized scores in 2019		
Took the test in 2020	0.092	0.089
	(0.0002)	(0.0002)
N	2,232,676	
**Panel B**: Standardized scores in 2020		
Remote learning	-0.222	-0.225
	(0.0002)	(0.0002)
N	6,142,212	
Grade fixed-effects	no	yes

Notes: Panel A restricts the sample to students who took the standardized test in Q4/2019, regressing standardized test scores on an indicator variable of whether students also took the standardized test in Q4/2020. Panel B restricts the sample to students who took standardized tests in Q1/2019, Q4/2019, Q1/2020, and Q4/2020. It estimates a differences-in-differences model comparing variation in outcomes between Q1- and Q4-2020 to that between Q1- and Q4-2019. The dependent variable is scores from quarterly standardized tests (*AAPs*), averaging math and Portuguese scores for that school quarter. Column (2) controls for grade fixed-effects. All columns are OLS regressions, with standard errors clustered at the school level. P-values computed from two-sided t-tests that each coefficient is equal to zero. * if p<0.1, ** p<0.05 and *** if p<0.01.

F. Non-parametric treatment effects by local disease activity

Supplementary Figure F.1 estimates heterogeneous treatment effects of remote learning on high dropout risk (Panel A) and standardized test scores (Panel B) by variation in per capita COVID-19 cases between Q1- and Q4-2020. In each panel, both variables are residualized with respect to all covariates that we observe and their interactions with a Q4 indicator (= 1 in the last school quarter, and 0 otherwise).

Figure S.F.1: Non-parametric heterogeneous treatment effects on educational outcomes by local COVID-19 cases

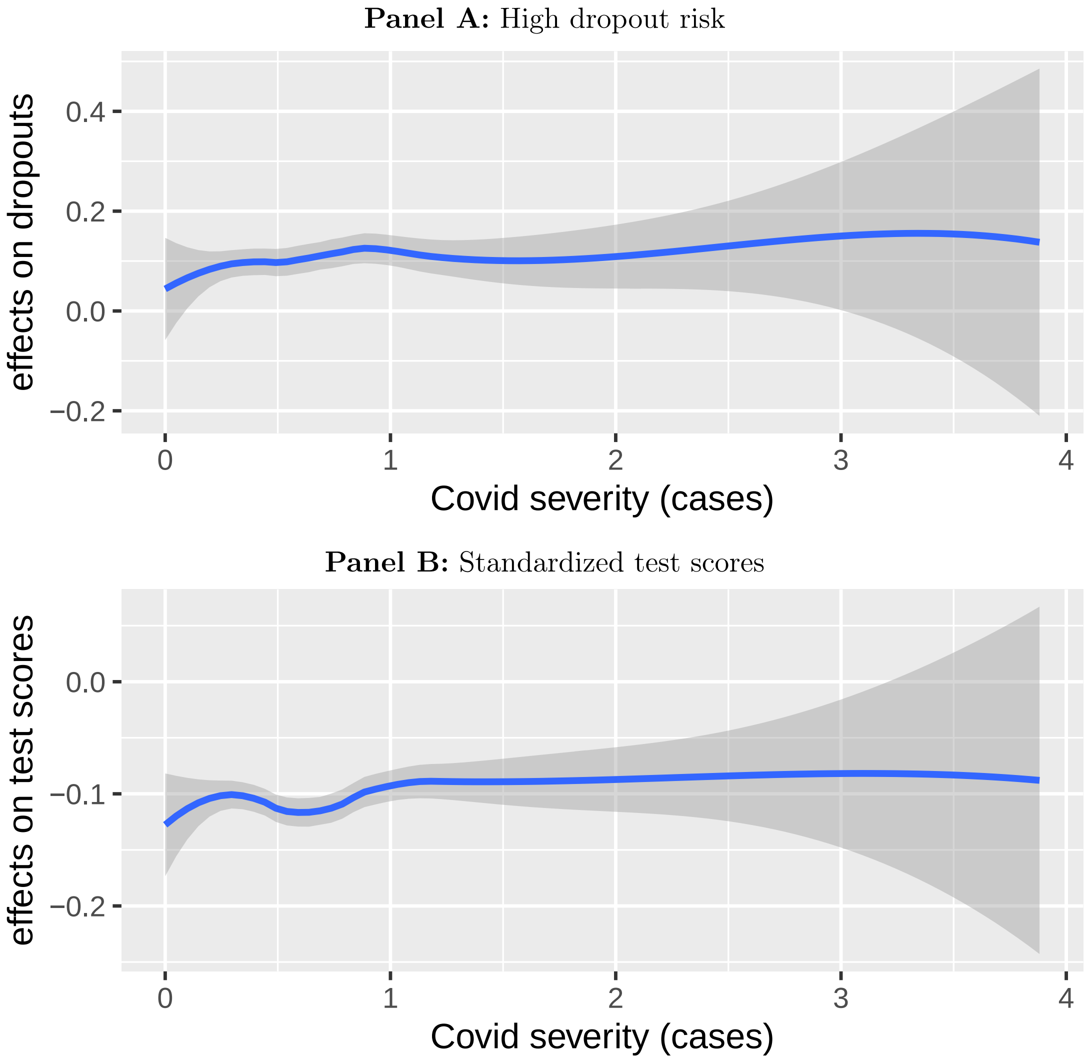

Notes: The blue line shows local polynomial regressions of treatment effects on high dropout risk and treatment effects on standardized test scores on municipal-level per capita COVID-19 cases x Q4 (labeled as *COVID severity*). The shaded gray area represents 95% confidence intervals for these estimates. Both variables were residualized with respect to a Q4-2020 indicator, all student and school characteristics, municipal-level per capita COVID-19 cases and deaths in each of the previous quarters, as well as interactions between student and school characteristics and per capita COVID-19 cases in each quarter. Estimates are local linear regressions with bandwidth = 0.8.

These results are not sensitive to the measure of pandemic severity. In Supplementary Figure F.2, we find almost identical results using COVID-19 related deaths:

Figure S.F.2: Non-parametric heterogeneous treatment effects on educational outcomes by local COVID-19 deaths

Panel A: High dropout risk

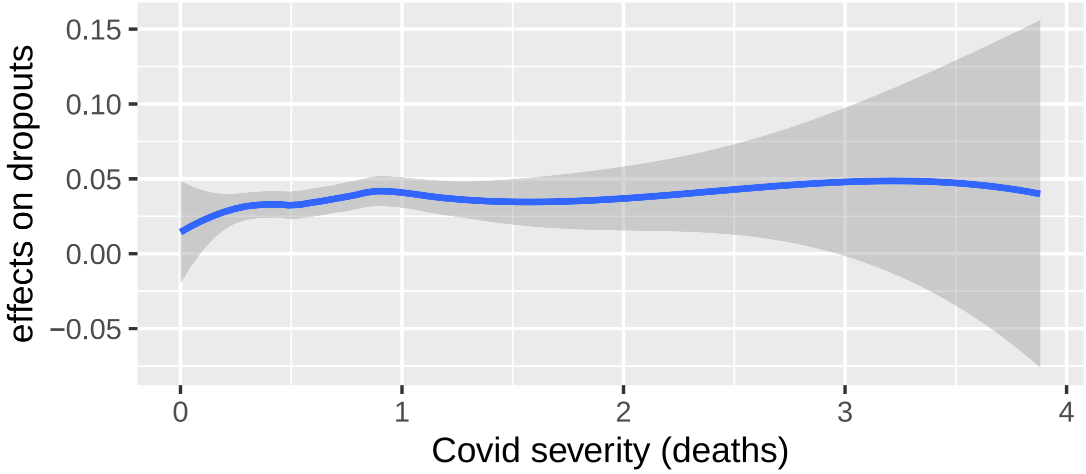

Panel B: Standardized test scores

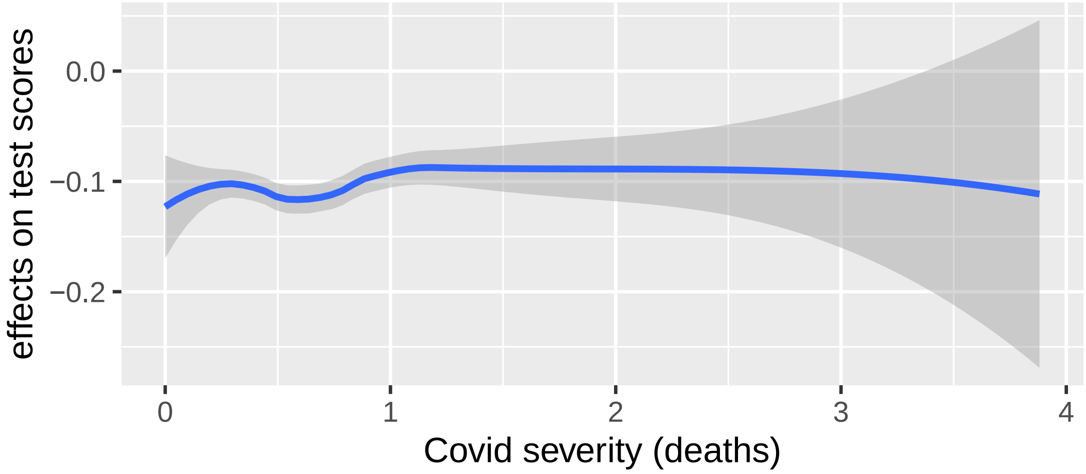

Notes: The blue line shows local polynomial regressions of treatment effects on high dropout risk and treatment effects on standardized test scores on municipal-level per capita COVID-19 deaths x Q4 (labeled as *COVID severity*). The shaded gray area represents 95% confidence intervals for these estimates. Both variables were residualized with respect to a Q4-2020 indicator, all student and school characteristics, municipal-level per capita COVID-19 cases and deaths in each of the previous quarters, as well as interactions between student and school characteristics and per capita COVID-19 cases in each quarter. Estimates are local linear regressions with bandwidth = 0.8.

G. The school reopening process

As discussed in the main text, some schools partially reopened to in-person activities at the end of 2020. In this section, we provide additional details on the reopening process. We provide more details in (*8*). São Paulo State authorized municipalities to reopen schools for optional activities (remedial classes for students lagging behind, and extra-curricular activities, such as psychological counselling) from September 8th to high-school students, and from October 7th to primary- and middle-school students. Regular in-person classes for high-school students were authorized to return from November 3rd. Only municipalities within health regions with stable pandemic conditions were allowed to return.

Municipalities had autonomy to decree whether schools could reopen, as long as safe reopening protocols were in place; in particular, all school staff had to wear personal protective equipment, alcohol had to be made available at the school gate, and attendance was limited (e.g. at 35% capacity in regions where the severity of the pandemic was high). The State Secretariat of Education estimates that 1,700 schools were in fact open for in-person activities and that at least 2 million students did go to school during that period.

The reopening process was staggered across municipalities. Supplementary Figure G.1 shows the cumulative distribution of municipalities which authorized schools to reopen over time.

Figure S.G.1: Cumulative distribution of school reopening authorization decrees in São Paulo State

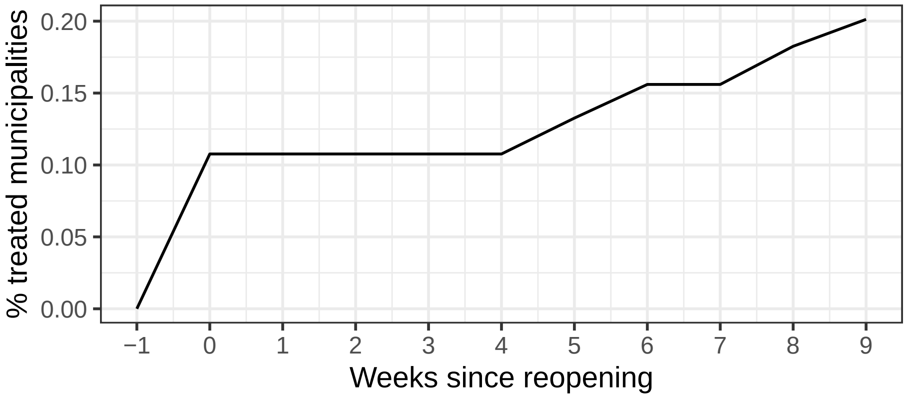

Notes: The figure shows the fraction of total municipalities in São Paulo state that allowed schools to return to in-person activities. The week of reference is the first week of October.

Naturally, the decision to reopen schools for in-person activities was not randomly assigned. External conditions mattered; in particular, municipalities in health regions with high disease activity *could not* issue authorization decrees before reaching a low enough threshold for COVID-19 cases and in-patient hospitalizations. Moreover, even among those that could, municipalities that authorized schools to reopen were not identical to those that did not. Supplementary Table G.1 provides descriptive statistics of the municipalities that reopened schools and those that did not. Municipalities that reopened schools had a lower number of new COVID-19 cases and deaths, were relatively poorer, and less populous.

Table S.G.1: Descriptive statistics in the baseline (end of September)

	Never treated	Ever treated	p-value difference
New cases per thousand	0.79	0.76	0.71
New deaths per thousand	0.03	0.02	0.23
Accumulated deaths per thousand	0.44	0.49	0.20
Income per capita	672.17	804.58	$<$0.001
Population (thousands)	38.65	200.37	0.09
Number of schools	19.41	67.94	0.03
Number of students (thousands)	7.21	34.34	0.55
School infrastructure	-0.01	0.00	0.88
Municipalities	514	129	

Notes: The table displays averages of several variables for municipalities that authorized schools to reopen for in-person classes in 2020 and those that did not. The third column show the p-value for the hypothesis that the means are equal between groups.

References

1. A. M. O. Dapeng Hu and Chong Wang and, A method of back-calculating the log odds ratio and standard error of the log odds ratio from the reported group-level risk of disease. *PLOS ONE.*

2. J. A. Hausman, J. Abrevaya, F. M. Scott-Morton, Misclassification of the dependent variable in a discrete-response setting. *Journal of Econometrics* **87** (1998).

3. B. D. Meyer, N. Mitaag, Misclassification in Binary Choice Models. *Working paper* (2016).

4. P. M.S., D. Nogueira, Análise da matriz de competência em uma aplicação real da Avaliação da Aprendizagem em Processo de Língua Portuguesa da Secretaria da Educação do Estado de São Paulo. *Revista de Estudos Linguísticos* (2017).

5. P. M.S., D. Nogueira, Analisando a ferramenta Avaliação de Aprendizagem em Processo da Secretaria da Educação Básica do Estado de São Paulo como método de ensino de língua materna. *Revista de Estudos Linguísticos* (2017).

6. *Mapas de Foco da BNCC*, `https://institutoreuna.org.br/projeto/mapas-de-foco-bncc`, Accessed: 2021-05-21.

7. *Plataforma de apoio à aprendizagem*, `https://apoioaaprendizagem.caeddigital.net/#!/funciona`, Accessed: 2021-05-21.

8. G. Lichand, C. Alberto Doria, J. Cossi, O. Leal Neto, Reopening Schools in the Pandemic Did Not Increase COVID-19 Incidence and Mortality in Brazil. *JAMA Health Forum* (2021).
