# Supplementary material for: The impacts of remote learning in secondary education during the pandemic in Brazil
Source: Nat Hum Behav. 2022 May 26;6(8):1079–86. doi: 10.1038/s41562-022-01350-6 (PMC9391221; doi:10.1038/s41562-022-01350-6)
Supplement: Supplementary file 2 — Reporting Summary [file 41562_2022_1350_MOESM2_ESM.pdf]

nature research

Corresponding author(s): Guilherme Lichand

Last updated by author(s): Mar 29, 2022

Reporting Summary

Nature Research wishes to improve the reproducibility of the work that we publish. This form provides structure for consistency and transparency in reporting. For further information on Nature Research policies, see our Editorial Policies and the Editorial Policy Checklist.

Statistics

For all statistical analyses, confirm that the following items are present in the figure legend, table legend, main text, or Methods section.

n/a	Confirmed	
☐	☒	The exact sample size (n) for each experimental group/condition, given as a discrete number and unit of measurement
☐	☒	A statement on whether measurements were taken from distinct samples or whether the same sample was measured repeatedly
☐	☒	The statistical test(s) used AND whether they are one- or two-sided<br>*Only common tests should be described solely by name; describe more complex techniques in the Methods section.*
☐	☒	A description of all covariates tested
☐	☒	A description of any assumptions or corrections, such as tests of normality and adjustment for multiple comparisons
☐	☒	A full description of the statistical parameters including central tendency (e.g. means) or other basic estimates (e.g. regression coefficient) AND variation (e.g. standard deviation) or associated estimates of uncertainty (e.g. confidence intervals)
☐	☒	For null hypothesis testing, the test statistic (e.g. F, t, r) with confidence intervals, effect sizes, degrees of freedom and P value noted<br>*Give P values as exact values whenever suitable.*
☒	☐	For Bayesian analysis, information on the choice of priors and Markov chain Monte Carlo settings
☒	☐	For hierarchical and complex designs, identification of the appropriate level for tests and full reporting of outcomes
☐	☒	Estimates of effect sizes (e.g. Cohen's d, Pearson's r), indicating how they were calculated

Our web collection on statistics for biologists contains articles on many of the points above.

Software and code

Policy information about availability of computer code

Data collection	We used an R studio server (version 3.4.4) to connect and extract sensitive data for this project in a secure cloud-based environment. Codes are available at https://github.com/Carlosalbertobdc/school-reopening.
Data analysis	We subsequently use the same R server (version 3.4.4) to conduct the data analysis. Codes are also available at https://github.com/Carlosalbertobdc/school-reopening.

For manuscripts utilizing custom algorithms or software that are central to the research but not yet described in published literature, software must be made available to editors and reviewers. We strongly encourage code deposition in a community repository (e.g. GitHub). See the Nature Research guidelines for submitting code & software for further information.

Data

Policy information about availability of data

All manuscripts must include a data availability statement. This statement should provide the following information, where applicable:

- Accession codes, unique identifiers, or web links for publicly available datasets
- A list of figures that have associated raw data
- A description of any restrictions on data availability

The data that support the findings of this study was made available by the São Paulo Education Secretariat (SEDUC-SP). Restrictions apply to the availability of these data due to students' personal identifiers. Data access requires an agreement with SEDUC-SP.

Field-specific reporting

Please select the one below that is the best fit for your research. If you are not sure, read the appropriate sections before making your selection.

☐ Life sciences ☒ Behavioural & social sciences ☐ Ecological, evolutionary & environmental sciences

For a reference copy of the document with all sections, see nature.com/documents/nr-reporting-summary-flat.pdf

Behavioural & social sciences study design

All studies must disclose on these points even when the disclosure is negative.

Study description	In this study, we provide a quasi-experimental quantitative assessment of the effects of school closures and reopening on students' standardized test scores and dropout risk.
Research sample	The study sample includes all students in secondary education in São Paulo State schools between 2018, 2019, and 2020.
Sampling strategy	We use data on the universe of State public school students.
Data collection	We only use administrative data that the São Paulo State Education Secretariat made available via a remote server.
Timing	We use administrative data collected from 2018 to 2020.
Data exclusions	We exclude observations with unavailable data on test scores or socioeconomic characteristics. This amounts to 8% of the full sample (1,135,526 observations).
Non-participation	We use data on the universe of State public school students.
Randomization	Students' were not randomly allocated into treatment and control groups.

Reporting for specific materials, systems and methods

We require information from authors about some types of materials, experimental systems and methods used in many studies. Here, indicate whether each material, system or method listed is relevant to your study. If you are not sure if a list item applies to your research, read the appropriate section before selecting a response.

Materials & experimental systems

n/a	Involved in the study	
☒	☐	Antibodies
☒	☐	Eukaryotic cell lines
☒	☐	Palaeontology and archaeology
☒	☐	Animals and other organisms
☒	☐	Human research participants
☒	☐	Clinical data
☒	☐	Dual use research of concern

Methods

n/a	Involved in the study	
☒	☐	ChIP-seq
☒	☐	Flow cytometry
☒	☐	MRI-based neuroimaging
